# Efficient Diesel Desulfurization by Novel Amphiphilic Polyoxometalate-Based Hybrid Catalyst at Room Temperature

**DOI:** 10.3390/molecules28062539

**Published:** 2023-03-10

**Authors:** Jie Zhao, Bingquan Wang, Rui Wang, Ivan V. Kozhevnikov, Korchak Vladimir

**Affiliations:** 1 School of Environmental Science and Engineering, Shandong University, Qingdao 266237, China; 2 School of Chemistry and Molecular Engineering, Qingdao University of Science and Technology, Qingdao 266042, China; 3 Department of Chemistry, University of Liverpool, Liverpool L69 7ZD, UK; 4 N. N. Semenov Federal Research Center of Chemical Physics, Russian Academy of Sciences, Moscow 119991, Russia

**Keywords:** desulfurization, diesel, polyoxometalate, oxidation catalysis

## Abstract

Amphiphilic hybrid catalysts were prepared by modifying [SMo_12_O_40_]^2−^ with tetrabutylammonium bromide (TBAB), 1-butyl-3-methylimidazole bromide (BMIMBr) and octadecyl trimethyl ammonium bromide (ODAB), respectively. The prepared catalysts were characterized by IR, XRD, SEM, TG and XPS. The desulfurization performance of the catalysts was investigated in model oil and actual diesel using hydrogen peroxide (H_2_O_2_) as an oxidant and acetonitrile as an extractant. All catalysts exhibited favorable activity for removing sulfur compounds at room temperature. Dibenzothiophene (DBT) can be nearly completely removed using SMo_12_O_40_^2−^-organic catalysts within a short reaction time. For different sulfur compounds, the [TBA]_2_SMo_12_O_40_ catalyst showed a better removal effect than the [BMIM]_2_SMo_12_O_40_ and [ODA]_2_SMo_12_O_40_ catalyst. The [TBA]_2_SMo_12_O_40_ dissolved in extraction solvent could be reused up to five times in an oxidative desulfurization (ODS) cycle with no significant loss of activity. The [BMIM]_2_SMo_12_O_40_ performed as a heterogeneous catalyst able to be recycled from the ODS system and maintained excellent catalytic activity. The catalysts showed a positive desulfurization effect in real diesel treatment. Finally, we described the ODS desulfurization mechanism of DBT using SMo_12_O_40_^2−^-organic hybrid catalysts. The amphiphilic hybrid catalyst cation captures DBT, while SMo_12_O_40_^2−^ reacts with the oxidant H_2_O_2_ to produce peroxy-active species. DBT can be oxidized to its sulfone by the action of peroxy-active species to achieve ODS desulfurization.

## 1. Introduction

Diesel is an important energy source and plays a vital role in industrial production [1,2]. Sulfur-containing compounds are one of the main pollutants in diesel, and its existence will bring many adverse effects. The combustion of sulfur-containing compounds produces SO_x_, which can cause air pollution and acid rain [3,4,5]. In addition, the presence of sulfur compounds in diesel can also poison the catalytic converter and corrode internal refinery components. As a result, countries around the world have implemented and proposed more stringent fuel standards [6]. Most countries require that the sulfur content in fuel cannot be higher than 10 ppm [7,8,9,10]. At present, fuel desulfurization technology is mainly divided into two categories: hydrodesulfurization (HDS) and non-hydrodesulfurization. HDS is the most widely used desulfurization technology in the industry [11,12]. HDS requires hydrogen consumption and high temperature and pressure operating conditions, which increase equipment input and economic costs [13,14]. The conventional catalytic HDS process has the least efficiency for the catalytic hydrodesulfurization of thiophene sulfides [15]. Therefore, the high input cost and poor removal effect of thiophene sulfides limit the development and application of HDS technology. Non-hydrodesulfurization technologies include extractive desulfurization (EDS), biological desulfurization (BDS), adsorption desulfurization (ADS), and oxidative desulfurization (ODS) technologies [16]. Among them, ODS is considered to be the industrial replacement technique for HDS with the most potential because of its mild reaction conditions, good removal effect with regard to thiophene sulfides, and low input costs [17]. ODS refers to the method in which sulfides are oxidized into corresponding sulfoxides/sulfones under the action of oxidants and catalysts and are removed from the oil by extraction, adsorption or distillation [18,19,20,21].

The choice of oxidant and catalyst has an important influence on ODS performance. Oxygen (O_2_), ozone (O_3_), hydrogen peroxide (H_2_O_2_), and organic peroxides are normally used to act as oxidants in ODS systems [3,17]. Among them, H_2_O_2_ is widely used in the ODS process due to its strong oxidizing ability and environmental friendliness [22,23,24,25]. Because of their unique molecular structure and excellent chemical properties, polyoxometalates (POMs) have always been hot materials in the field of catalysis [26,27,28]. Researchers have found that changing the heteroatom in Keggin heteropoly anions leads to different redox properties. Himeno et al. performed a systematic analysis of the voltammetric properties of Keggin-type polyoxometalate complexes (α-[XMo_12_O_40_]^n−^ (X = S, P, As, Si, Ge; *n* = 2–4) and α-[XW_12_O_40_]^n−^ (X = S, P, As, Si, Ge, B, Al; *n* = 2–5) based on alkalinity. The results show that the basicity of the Keggin anions is in the order of [SMo_12_O_40_]^2−^ ≈ [XW_12_O_40_]^3−^ (X = P, As) << [XMo_12_O_40_]^3−^ (X = P, As) < [XW_12_O_40_]^4−^ (X = Si, Ge) << [XMo_12_O_40_]^4−^ (X = Si, Ge) < [XW_12_O_40_]^5−^ (X = B, Al) [29]. Wedd’ s group demonstrated that the sulfo-polyoxometalate anion clusters [S_2_W_18_O_62_]^4−^, [S_2_Mo_18_O_62_]^4−^ and [SMo_12_O_40_]^2−^ can be activated photochemically to oxidize the organic substrates benzyl alcohol, ethanol, and (−)-menthol [30].They then subsequently found in reduction studies of Mo and W polyoxometalate anions in both ionic liquid and conventional solvent (electrolyte) media that Mo polyoxometalates are always easier to reduce than their W counterparts, and that reduced W polyoxometalates are more stable than their isostructural Mo analogues (these differences are possibly associated with the higher electron affinity of Mo(VI) sites and the higher proton affinity of -O- sites in Mo polyoxometalates, which makes the Mo moieties easier to reduce and also more prone to attack by protons than their W counterparts) [31]. However, when POMs are employed as ODS catalysts, the fact that POMs are insoluble in the organic oil phase hinders the effective contact between the catalyst and sulfur compounds, affecting the ODS efficiency [32,33]. In order to solve this problem, some scholars proposed the addition of an extractant into the ODS system [34,35]. The lipophilicity oil phase and hydrophilic extraction phase form a two-phase system in which the POM is dissolved in the extraction phase. Sulfur compounds in the oil phase are extracted into the aqueous phase, and react with oxidants under catalytic action to form corresponding polar sulfones. Although the problem of insolubility of the POM catalyst can be solved by adding extractant into the ODS system, the mass transfer resistance between the oil phase and the extraction phase still hinders the desulfurization efficiency and reaction rate.

For this reason, many researchers have proposed to introduce amphiphilic component into the POM to reduce the mass transfer resistance between the two phases. The amphiphilic POM-based catalyst can make use of the lipophilicity of the cation to make the sulfides in the oil and the oxidation active center have better contact so that the desulfurization reaction can proceed faster and better [36,37]. POMs can be modified by organic units such as quaternary ammonium salts, ionic liquids, oligomers, etc. [38]. To date, a lot of work on amphiphilic POM-based catalysts has been reported. Li et al. synthesized the amphiphilic catalyst by the combination of quaternary ammonium salt and phosphotungstate, and studied the effect of different quaternary ammonium cations on the performance of the catalyst [39]. The results showed that [(C_18_H_37_)_2_N(CH_3_)_2_]_3_[PW_12_O_40_] had excellent activity. When the temperature was 30 °C, the desulfurization rate could reach 100% after 80 min of reaction. In 2006, Lv et al. reported another amphiphilic catalyst [(C_18_H_37_)_2_N(CH_3_)_3_]_4_[H_2_NaPW_10_O_36_] [40], which can complete the highly active catalytic oxidation reaction in an ODS system with high sulfur content. Wang’ s group prepared three hybrid POM materials [HPMo][TMAC]_2_, [HPMo][DTAC]_2_, and [HPMo][HTAC]_2_ by using modified phosphomolybdic acid (HPMo) with tetramethylammonium chloride (TMAC), dodecyltrimethylammonium chloride (DTAC), and cetyltrimethylammonium chloride (HTAC). Under the condition of H_2_O_2_ as the oxidant, the catalytic activity was compared in the ODS system. When [HPMo][HTAC]_2_ was used as a catalyst, the conversion rate of dibenzothiophene (DBT) reached 96% at 60 °C for 180 min. The reason that [HPMo][HTAC]_2_ has better catalytic performance is that the longer hexadecyl chain in the structure is not only more beneficial to the wrapping of DBT, but is also beneficial to the formation of a stable emulsion system containing a high DBT concentration [41]. Susana et al. reported three organic-inorganic hybrid catalysts-based [PW_11_Zn(H_2_O)O_39_]^5−^ [42]. As a phase transfer agent, the quaternary ammonium cation can promote the effective mass transfer between the oil phase and the extraction phase, and improve the desulfurization rate. Under the action of the catalyst processing tetrabutylammonium bromide cation, the reaction has a favorable removal effect on different sulfur compounds. Li et al. studied amphiphilic catalysts with a core-shell structure [15]. The hydrophilic core is composed of phosphotungstste (PWO) clusters as the catalytic center, and the lipophilic shell consisted of long chain alkyl-imidazole or pyridine cations. DBT could be completely oxidized at 40 °C within 40 min under the catalysis of [C_16_MIM]_3_PWO. This method is useful for actual diesel desulfurization. Amphiphilic ODS catalysts are frequently reported [37,43]. The design of an amphiphilic ODS catalyst with high activity, selectivity, and recyclability is a research hotspot in the field of fuel desulfurization.

In this paper, we studied three kinds of amphiphilic hybrid catalysts based on [SMo_12_O_40_]^2−^. The polyacid anion [SMo_12_O_40_]^2−^ is a saturated α-Keggin type POM. In the POM, all of the Mo exhibit the geometric environment of the {MoO_6_} octahedron. The central S atom is surrounded by eight oxygen atoms connected to it, forming a small cube at the center of the entire polyacid. The whole α-Keggin POM is composed of 12 {MoO_6_} octahedrons connected by common edges and common angles, and then connected with the center {SO_4_} unit at common angles. The S element in [SMo_12_O_40_]^2−^ anion appears as the sulfate species. POM with the S^6+^ heteroatom is rare. The S^6+^ heteroatom helps to disperse the negative charges on the surface of the POM and makes the POM more stable. Different hybrid catalysts were prepared by modifying [SMo_12_O_40_]^2−^ with a tetrabutylammonium (TBA), 1-butyl-3-methylimidazolium (BMIM), and octadecyldimethulammonium (ODA) cation. The catalytic performance of catalysts was evaluated in model oil and actual diesel using H_2_O_2_ as the oxidant and acetonitrile as the extractant. Various reaction conditions that affect desulfurization efficiency are optimized in the ODS process and the recovery performance of catalysts was investigated. To the best of our knowledge, this is the first report on the application and optimization of the SMo_12_O_40_^2−^-organic hybrid catalysts in the ODS system.

## 2. Results and Discussion

### 2.1. Characterization of Catalysts

**Figure 1:**
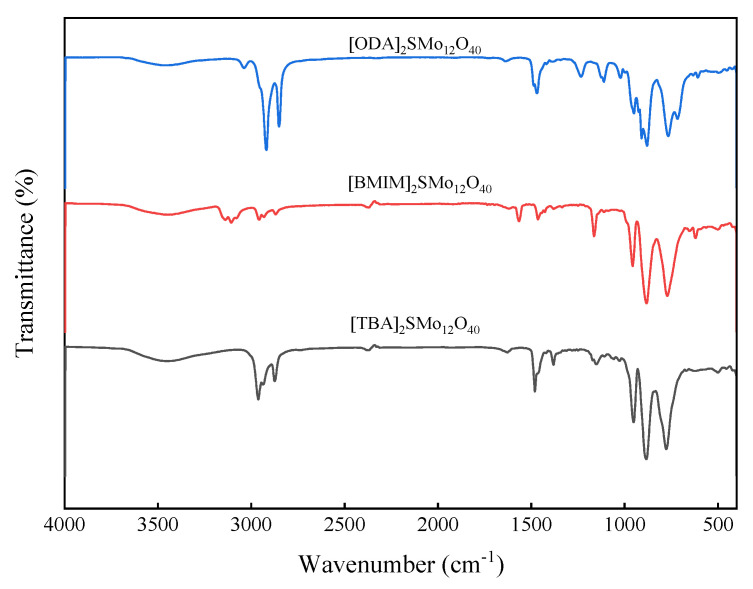
IR spectra of the catalysts.

**Figure 2:**
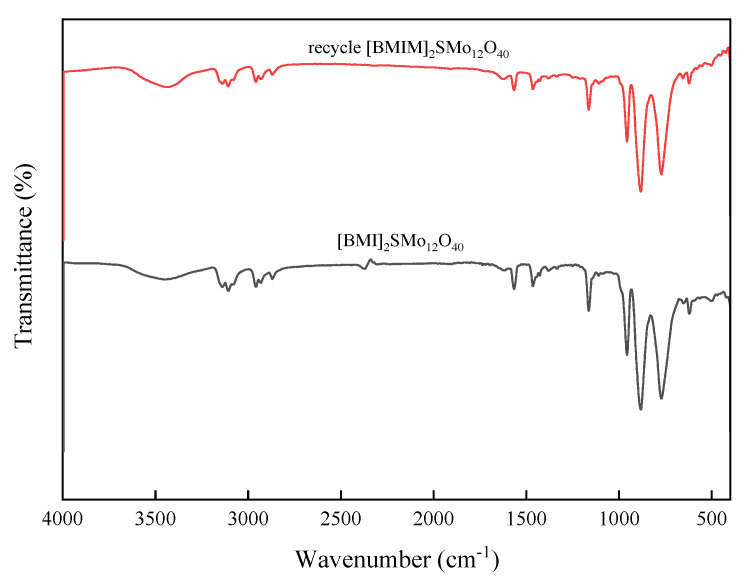
IR spectra of the [BMIM]_2_SMo_12_O_40_ catalyst before and after recovery.

The hybrid catalysts based on [SMo_12_O_40_]^2−^ were characterized by IR. The characteristic absorption peaks of [SMo_12_O_40_]^2−^ with a Keggin structure are shown in Figure 1. Due to the difference of organic cations, the characteristic peak of the polyanion has shifted to different degrees. The characteristic bands of S-O in catalyst structures occurs at 1151, 1163 and 1111 cm^−1^, respectively. The peaks of wave number at 952, 957 and 950 cm^−1^ are attributed to Mo=O. The (Mo-O-Mo) symmetric or asymmetric vibration approximately appears in 885–880 cm^−1^ and 778–765 cm^−1^ [44]. These characteristic peaks indicate the presence of [SMo_12_O_40_]^2−^–polyoxometalate. In the [TBA]_2_SMo_12_O_40_ catalyst, the peaks at 2962, 2874 and 1481 cm^−1^ belong to the vibration of the TBA cation [45,46]. In the [BMIM]_2_SMo_12_O_40_ catalyst, the peaks observed at 3144 and 3108 cm^−1^ are attributed to the imidazole ring’s hydrogen. The stretching absorption peak of n-butyl’s hydrogenate can be assigned as 2957 and 2926 cm^−1^. The characteristic peaks of the imidazole ring are observed at 1566 and 1465 cm^−1^ [43,47]. In the [ODA]_2_SMo_12_O_40_ catalyst, the vibration peaks at 2918, 2851 and 1469 cm^−1^ are characteristic of an ODA cation structure [27]. Figure 2 shows the IR spectra of the [BMIM]_2_SMo_12_O_40_ catalyst before and after recovery. The IR of the recovered [BMIM]_2_SMo_12_O_40_ catalyst showed that no obvious characteristic peak was destroyed, indicating that the structure of the recovered catalyst remained intact.

**Figure 3:**
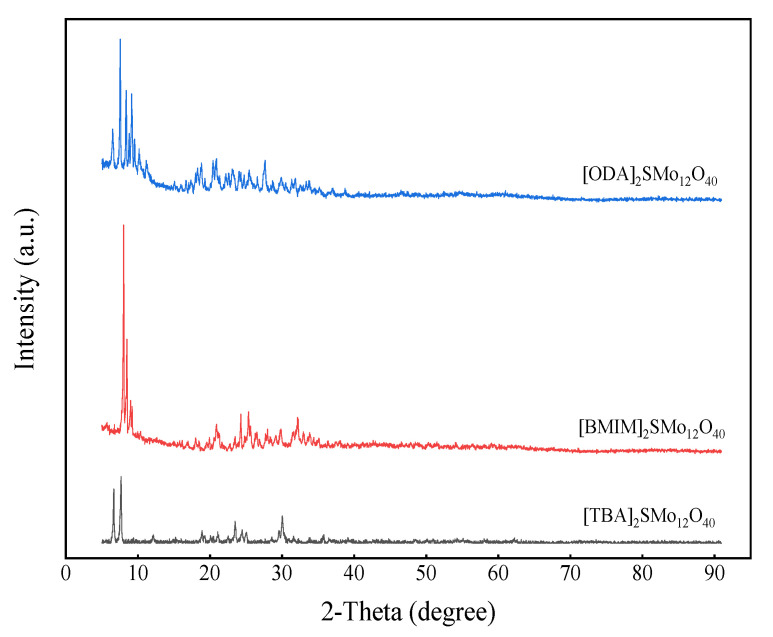
XRD patterns of the catalysts.

The XRD patterns of catalysts are shown in Figure 3. The strong characteristic peak appeared in the 2θ range of 6.5°–10°, indicating the ordering of the Keggin bulk structure of the polyoxoanion [48]. Due to the introduction of different organic cations, some weak characteristic peaks appeared in different 2θ range. The intensity of characteristic peaks in [TBA]_2_SMo_12_O_40_ are weaker than [BMIM]_2_SMo_12_O_40_ and [OTA]_2_SMo_12_O_40_, indicating that the [SMo_12_O_40_]^2−^ group is finely dispersed with the mixing of TBAB [41].

**Figure 4:**
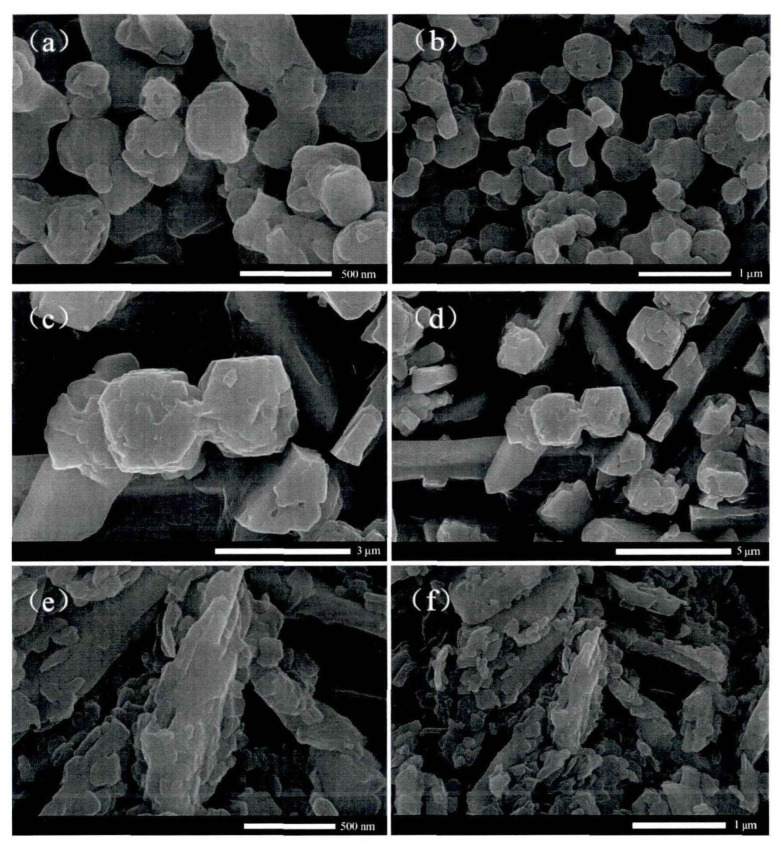
SEM images of (**a**,**b**) [TBA]_2_SMo_12_O_40_, (**c**,**d**) [BMIM]_2_SMo_12_O_40_, and (**e**,**f**) [ODA]_2_SMo_12_O_40_ catalysts.

SEM images of the prepared catalysts at different magnifications are shown in Figure 4. It can be seen from the SEM image that [SMo_12_O_40_]^2−^ performs as a small spherical particle with an irregular surface. The TBA cation component is in the shape of a short rod and is connected with the polyacid anion. The overall structure of the amphiphilic [TBA]_2_SMo_12_O_40_ catalyst is relatively loose. [BMIM]_2_SMo_12_O_40_ is composed of larger particles and has long rod-shaped ionic liquid components. The overall structure of [ODA]_2_SMo_12_O_40_ is relatively compact, and its surface is covered with a waxy layer because of the long-chain alkyl substitutes. [ODA]_2_SMo_12_O_40_ is a lamellar structure, and the active center [SMo_12_O_40_]^2−^ is attached to its surface.

**Figure 5:**
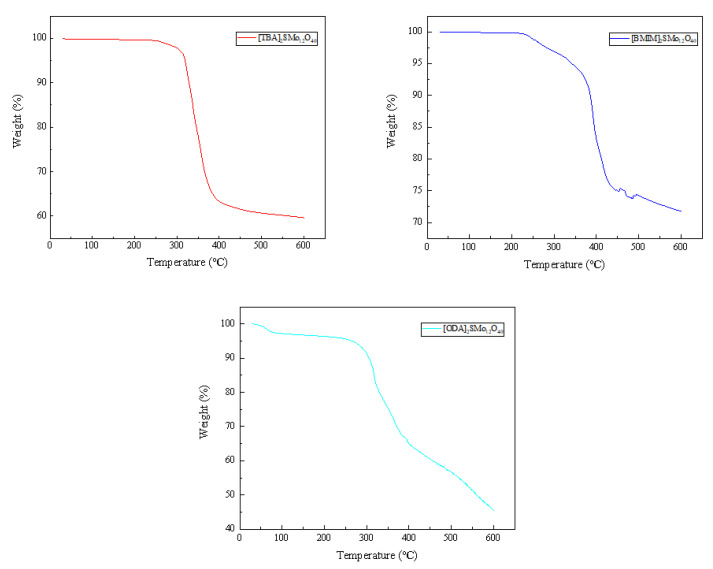
Thermogravimetric analysis of the catalysts.

The thermal stability of hybrid catalysts was studied by thermogravimetric analysis. The obtained results are shown in Figure 5. [TBA]_2_SMo_12_O_40_ and [BMIM]_2_SMo_12_O_40_ have weight loss regions between 240–600 °C and 220–600 °C, respectively. This demonstrates that [TBA]_2_SMo_12_O_40_ and [BMIM]_2_SMo_12_O_40_ begin to decompose. The [OTA]_2_SMo_12_O_40_ catalyst showed two weight loss areas. The first small weight loss of 2.5% observed between 36 and 78 °C is attributed to the removal of physically adsorbed water. The weight loss region (~50.7%) between 220–600 °C is assigned to the [OTA]_2_SMo_12_O_40_ that is beginning to decompose. The results of thermogravimetric analysis show that the decomposition temperature of the catalysts was higher than the reaction temperature of this experiment.

**Figure 6:**
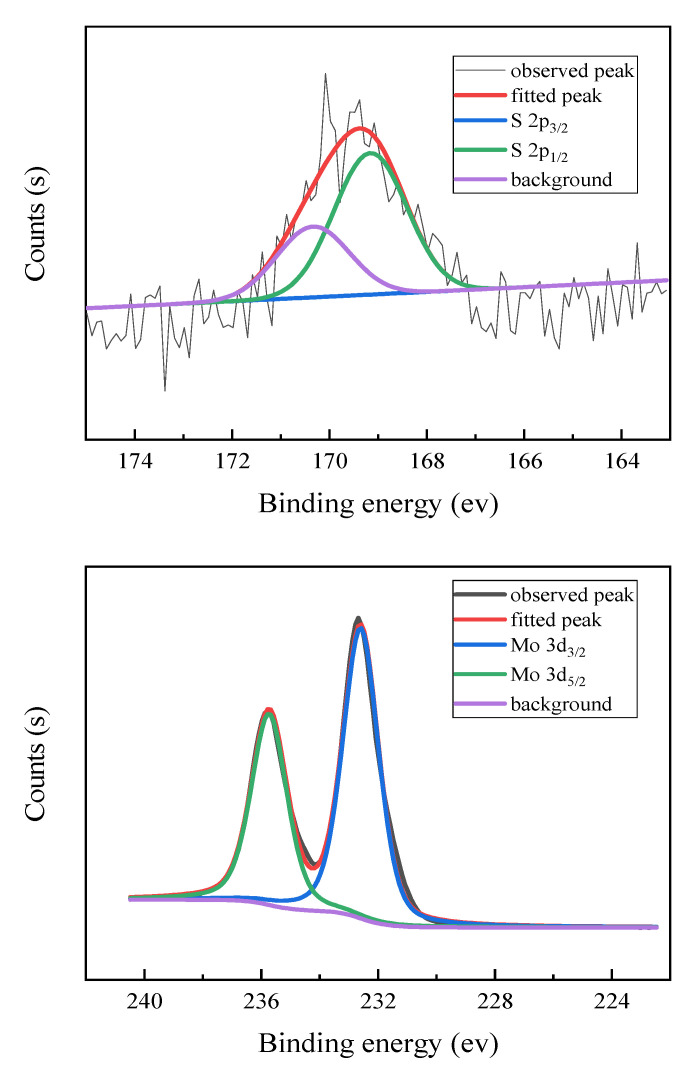
XPS spectra of [TBA]_2_SMo_12_O_40_ catalyst.

In order to determine the state of elements in the catalyst, an XPS characterization was performed. The results obtained are shown in Figure 6. Through the spectroscopy of Mo element in the [TBA]_2_SMo_12_O_40_ catalyst, it can be found that the binding energy of Mo 3d_5/2_ appears at 232.7 eV, indicating that the small peak is attributed to Mo^6+^ [49]. In the S 2P spectra of the [TBA]_2_SMo_12_O_40_ catalyst, absorption peaks of S 2p_3/2_ and S 2P_1/2_ were observed at 169.18 and 170.36 eV, and are attributed to the sulfate species contribution [50,51]. The [SMo_12_O_40_]^2−^ with S^6+^ heteroatom has an oxidizing ability.

### 2.2. Optimization of ODS System

The optimization in the ODS system was performed with the [TBA]_2_SMo_12_O_40_ catalyst. Various factors affecting desulfurization efficiency were investigated, including catalyst dosage, oxygen/sulfur molar ratio, and reaction temperature.

**Figure 7:**
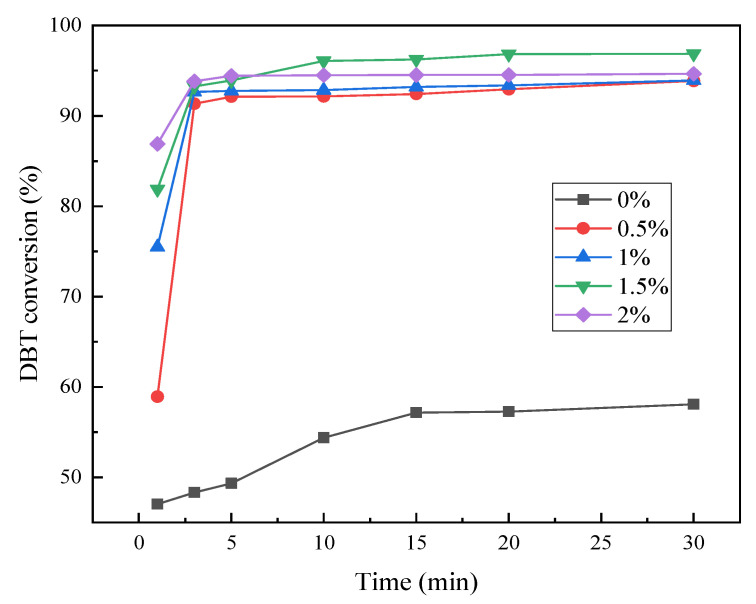
Influence of catalyst dosage on the conversion of DBT.

**Figure 8:**
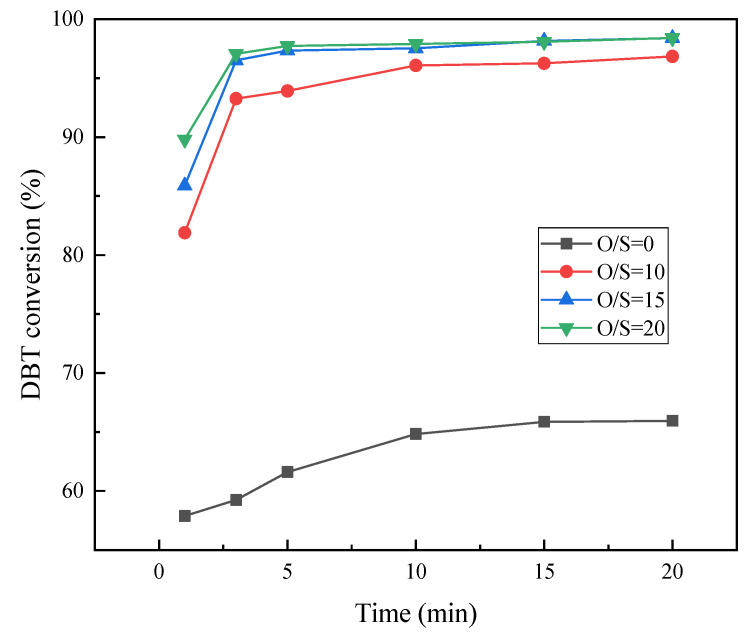
Influence of O/S molar ratio on the conversion of DBT.

The effect of different catalyst dosages (catalyst dosage to the mass ratio of model oil) on the ODS efficiency were shown in Figure 7. The experimental conditions were set to an initial sulfur content of 500 ppm, an O/S molar ratio of 10, and a temperature of 60 °C. As shown in Figure 8, when no catalyst is added, the DBT removal rate is only 58.62%, depending on the extraction of acetonitrile and the oxidation capacity of H_2_O_2._ In the presence of the catalyst, the desulfurization rate exceeded 91% when the reaction only proceeded for 3 min. In the initial stage of the reaction, the increase in the catalyst dosage is beneficial in improving the reaction rate. When the amount of catalyst increased from 0.21 g to 0.63 g, the desulfurization rate gradually increased. When the catalyst dosage was 0.84 g, the desulfurization rate decreased compared with that of 0.63 g. The desulfurization reaction basically reached a stable desulfurization effect in 10 min. When the catalyst dosage was 0.63 g, the best desulfurization rate (96.1%) could be obtained within 10 min. Therefore, the optimal catalyst dosage was determined to be 0.63 g (the catalyst dosage accounted for 1.5 wt% of the model oil quality).

The O/S molar ratio plays an important role in the desulfurization effect. Reactive oxygen molecules come from H_2_O_2._ According to the chemical equation of the DBT oxidation reaction, 1 mol DBT can be oxidized to the corresponding sulfone by 2 mol H_2_O_2_. However, due to the side reaction of self-decomposition of H_2_O_2_ in the reaction process, a larger amount of oxidant is often needed in the experiment. The influence of the O/S molar ratio was studied at 60 °C, using the model oil with an initial sulfur content of 500 ppm and a catalyst dosage of 0.63 g. The DBT conversion rates at different O/S molar ratios are shown in Figure 8. In the absence of oxidant, a desulfurization rate of 65.94% could be obtained within 20 min. With the addition of the oxidant, the desulfurization rate increased rapidly in a short time. The O/S molar ratio is directly proportional to the desulfurization rate and the reaction rate. When the O/S molar ratio was 20, the maximum desulfurization rate reached 98.40%, which was basically consistent with the desulfurization effect when the O/S molar ratio was 15 (desulfurization rate: 98.39%). Considering economic costs and energy utilization, the optimal O/S molar ratio was set to 15 for the following experiments.

**Figure 9:**
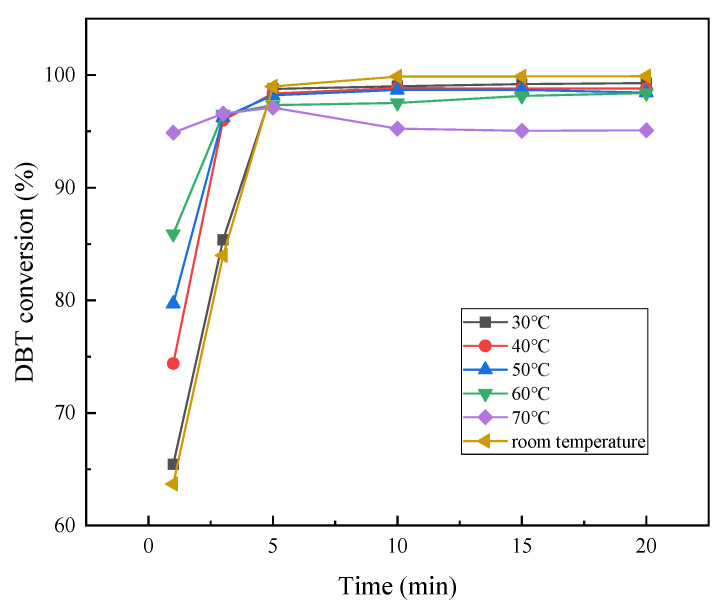
Influence of temperature on conversion of DBT.

Figure 9 shows the conversion rate of DBT at different temperatures, maintaining the other experimental conditions (the model oil with an initial sulfur content of 500 ppm, a catalyst dosage of 0.63 g and an O/S molar ratio of 15). The temperature increase is beneficial to increasing the reaction rate within 5 min. The DBT oxidation reaction at different temperatures reached equilibrium in 10 min. It is worth noting that a lower temperature can often achieve a higher desulfurization efficiency. The reason is that the rising temperature accelerates the decomposition of H_2_O_2_, which is not conducive to the continued progress of the DBT oxidation reaction, leading to less sulfur removal. Under the condition that the initial sulfur content is 500 ppm, O/S = 15, and the dosage of catalyst accounts for 1.5 wt% of the model oil quality, the DBT conversion rate reached 99.89% within 10 min at room temperature, achieving an ultra-fast and efficient desulfurization process. Desulfurization at room temperature can greatly reduce the economic cost and ensure fuel quality. Hence, the optimal reaction temperature is room temperature.

### 2.3. Comparison of Desulfurization Performance of Three Hybrid Catalysts Based [SMo_12_O_40_]^2−^

**Figure 10:**
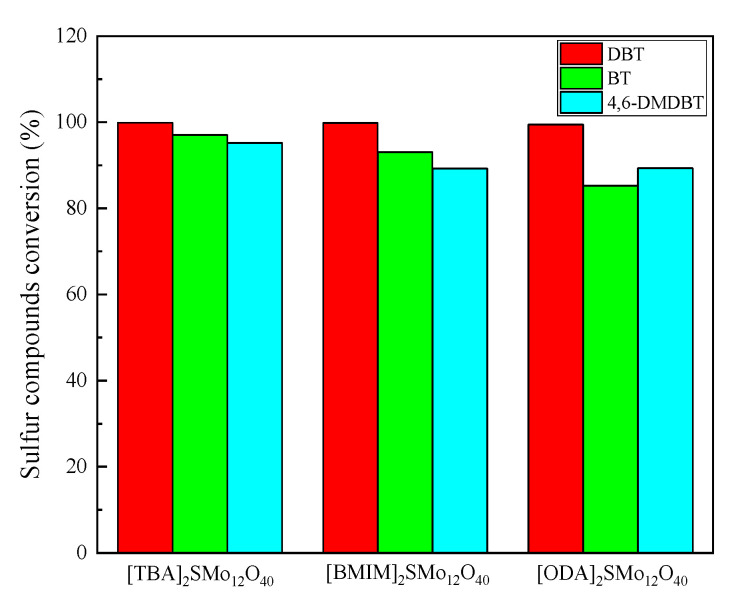
Desulfurization performance of catalysts for different sulfur compounds.

Under the above optimized reaction conditions, the desulfurization performance of the [TBA]_2_SMo_12_O_40_ catalyst for different sulfur compounds (DBT, BT and 4,6-DMDBT) was investigated (Figure 10). For the removal of DBT, a high conversion rate of 99.89% was achieved when the reaction was carried out for 10 min. BT and 4,6-DMDBT are sulfur compounds that are more difficult to oxidize than DBT. Under the catalysis of [TBA]_2_SMo_12_O_40_, the removal rates of BT and 4,6-DMDBT reached 97.06% and 95.20%, respectively, when the reactions were carried out for 90 min. In a comparison of the ODS activity of different catalysts in a previous work and in this study (Table 1), the [TBA]_2_SMo_12_O_40_ catalyst was shown to have a generally excellent desulfurization performance for different sulfides. The desulfurization performance of [BMIM]_2_SMo_12_O_40_ and [ODA]_2_SMo_12_O_40_ was also investigated by using the previous optimized reaction conditions (Figure 10). When [BMI]_2_SMo_12_O_40_ is used as a catalyst, the DBT removal rate can reach 99.81% within 15 min at room temperature. After the reactions proceeded for 90 min, BT and 4,6-DMDBT reached 93.05% and 89.21% conversion, respectively. Using an [ODA] _2_SMo_12_O_40_ catalyst, a 99.47% DBT removal rate was reached at 60 min. The conversion rates for BT and 4,6-DMDBT can be reached at 85.24% and 89.35% levels within 2 h, respectively.

The desulfurization effect of three organic-inorganic hybrid catalysts for different sulfides follows the following sequence: [TBA]_2_SMo_12_O_40_ > [BMIM]_2_SMo_12_O_40_ > [ODA]_2_SMo_12_O_40_. The [TBA]_2_SMo_12_O_40_ catalyst showed the best desulfurization efficiency. The catalytic activity of POMs-based hybrid catalysts is affected by the linked organic cations. Li et al. prepared a series of surfactant-type decatungstates catalysts [23]. They pointed out that the increase of the carbon chain length of the quaternary ammonium cation was beneficial to the enhancement of the activity of the catalyst. Zhuang et al. synthesized three molybdovanadophosphoric POM-based catalysts by contacting H_5_PMo_10_V_2_O_40_ with different ionic liquids [57]. They also reached a conclusion: The removal efficiency of the sulfur compound improved with the increase of the alkyl chain length of the catalyst. However, Lu et al. mentioned that quaternary ammonium cations with too long carbon chains may cause steric effects, reducing catalytic activity [49]. In our work, we observed differences in the solubility of three hybrid catalysts containing different cations in the acetonitrile. [TBA]_2_SMo_12_O_40_ is fully soluble in acetonitrile, allowing more effective contact between sulfur-containing compounds and oxidizing active substances, thereby obtaining a high desulfurization efficiency. [BMI]_2_SMo_12_O_40_ and [ODA]_2_SMo_12_O_40_ are insoluble in the oil phase, as isacetonitrile. Under the condition of magnetic stirring, [BMI]_2_SMo_12_O_40_ was evenly dispersed in the acetonitrile, while [ODA]_2_SMo_12_O_40_ showed an agglomeration phenomenon. The poor solubility of [ODA]_2_SMo_12_O_40_ is the main reason for its low desulfurization efficiency.

Under the catalysis of [TBA]_2_SMo_12_O_40_ and [BMIM]_2_SMo_12_O_40_, the activities of the three sulfur compounds all followed this sequence: DBT > BT > 4,6-DMDBT, which is consistent with the report by Li et al. [58]. However, 4,6-DMDBT showed a higher oxidation activity than BT when [ODA]_2_SMo_12_O_40_ acted as a catalyst. The activity of sulfur compounds is mainly related to electron density around the sulfur atom and steric hindrance. According to Otsuki’s report [59], the electron densities of sulfur atoms in DBT, BT and 4,6-DMDBT are 5.758, 5.739 and 5.760, respectively. The higher the electron density of sulfur atoms in sulfur compounds, the easier it is apt to be oxidized, so DBT is easier to remove than BT. However, the structure of 4,6-DMDBT contains two methyl groups. The effect of steric hindrance makes the removal process of 4,6-DMDBT more difficult. The [ODA]_2_SMo_12_O_40_ catalyst with the long alkyl chain has greater ease in wrapping up the larger molecule of 4,6-DMDBT close to the active center [41]. Hence, 4,6-DMDBT had a better removal effect than BT when using the [ODA]_2_SMo_12_O_40_ catalyst.

### 2.4. Catalyst Recovery

As excellent ODS catalysts, the recovery performance of [TBA]_2_SMo_12_O_40_ and [BMI]_2_SMo_12_O_4_ was investigated.

**Figure 11:**
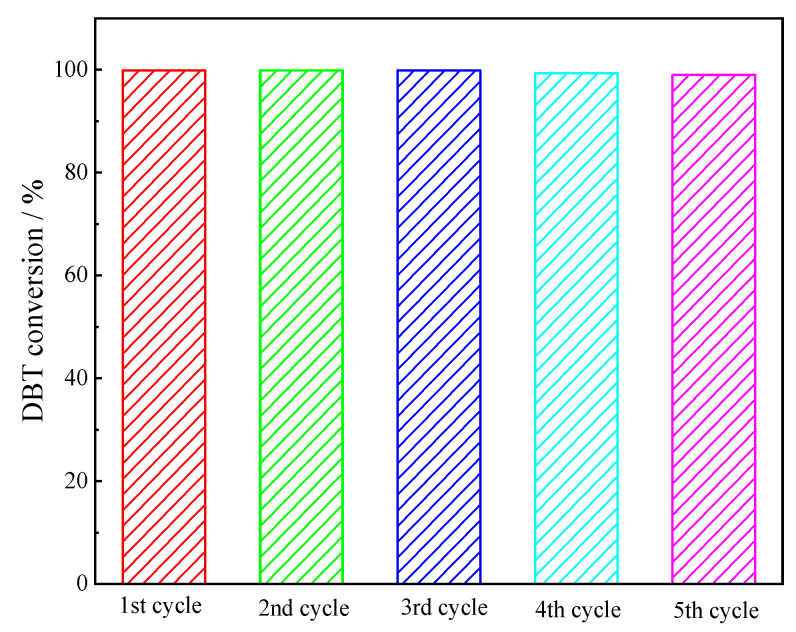
Desulfurization performance of reused [TBA]_2_SMo_12_O_40_ catalyst.

After the desulfurization reaction, [TBA]_2_SMo_12_O_40_ was completely dissolved in the acetonitrile phase and could not be separated from the ODS system as a solid. However, the acetonitrile extraction solution containing the dissolved catalyst could be reused for the removal of sulfur compounds. After each reaction, the upper low-sulfur oil was removed, and the fresh model oil with an initial sulfur content of 500 ppm and H_2_O_2_ (O/S = 15) were added to start a new round of ODS. The reaction time was set to 10 min. After five consecutive cycles of this, there was no apparent loss of catalyst activity. Figure 11 shows the desulfurization effect of the recovered [TBA]_2_SMo_12_O_40_ catalyst (99.89%, 99.89%, 99.89%, 99.32% and 99.01% of DBT conversion rate after 10 min for the first, to the fifth ODS cycle, respectively). The result showed that the [TBA]_2_SMo_12_O_40_ catalyst dissolved in acetonitrile still had an excellent catalytic activity after repeated use.

**Figure 12:**
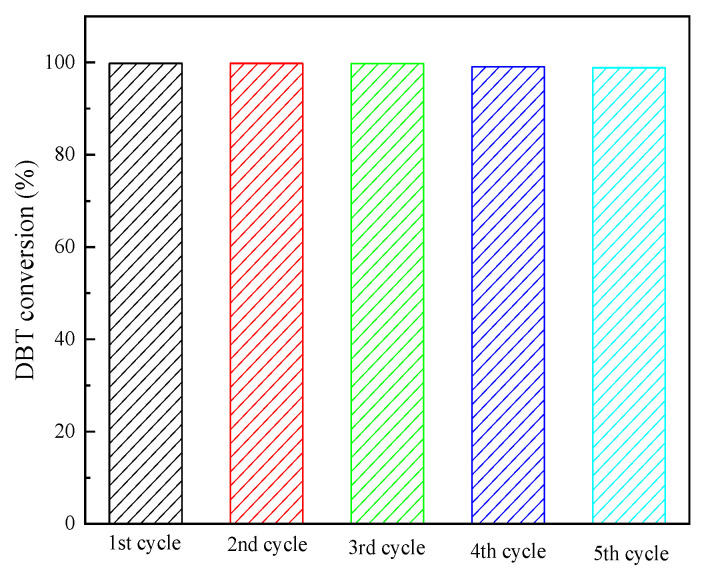
Desulfurization performance of the recycled [BMIM]_2_SMo_12_O_40_ catalyst.

[BMIM]_2_SMo_12_O_40_ performs heterogeneous catalysis in an ODS system. At the end of the reaction, the catalyst deposited on the bottom of the container was filtered, washed with distilled water, and dried in an oven. The desulfurization performance of the recovered [BMI]_2_SMo_12_O_40_ catalyst was evaluated in the optimized desulfurization system. After 15 min of reaction, the DBT conversion rate only reached 87%. This desulfurization result is lower than the desulfurization rate that was obtained during the initial catalysis. However, when the reaction time was extended to 45 min, the desulfurization rates obtained in five ODS recovery experiments were 99.81%, 99.81%, 99.78%, 99.07% and 98.87%, respectively (Figure 12). This is not significantly different from the initial desulfurization rate. Moreover, the IR of the catalyst before and after the recovery were similar, which indicated that the structure of the catalyst was not destroyed during the catalytic desulfurization reaction. Therefore, the [BMIM]_2_SMo_12_O_40_ catalyst has excellent recovery activity.

### 2.5. Oxidative Desulfurization of Real Diesel

The desulfurization performance of [TBA]_2_SMo_12_O_40_ and [BMIM]_2_SMo_12_O_40_ catalysts were evaluated in real diesel. Due to the complex and diverse components of real diesel, the removal effect of sulfur compounds is affected. The initial sulfur content of real diesel is 514.53 ppm. The reaction conditions are set as the amount of catalyst being 0.63 g (the amount of catalyst accounts for 1.5 wt% of the simulated oil quality), O/S = 15, and at room temperature. Using [TBA]_2_SMo_12_O_40_ as a catalyst, the sulfur content of real diesel was reduced from 514.53 ppm to 93.17 ppm after 150 min of reaction (Table 2). In order to further remove the sulfur compounds, the oil after the reaction was collected for the second ODS treatment, and the sulfur content in the oil was reduced to 20.18 ppm after 15 min. The total desulfurization rate of the [TBA]_2_SMo_12_O_40_ catalyst reached 96.08% after two ODS treatments. Under the catalysis of [BMIM]_2_SMo_12_O_40_, the sulfur content in the oil was decreased from 514.53 ppm to 41.52 ppm by two ODS treatments, maintaining the same reaction conditions (the reaction time of the first and second ODS process is 150 min and 60 min, respectively) (Table 2). The total desulfurization rate of the [BMIM]_2_SMo_12_O_40_ catalyst reached 91.93% after two ODS treatments. Hence, [TBA]_2_SMo_12_O_40_ and [BMIM]_2_SMo_12_O_40_ have favorable application prospects as catalysts.

### 2.6. The Mechanism of Oxidative Desulfurization Using SMo_12_O_40_^2−^-Organic Hybrid Catalysts

**Figure 13:**
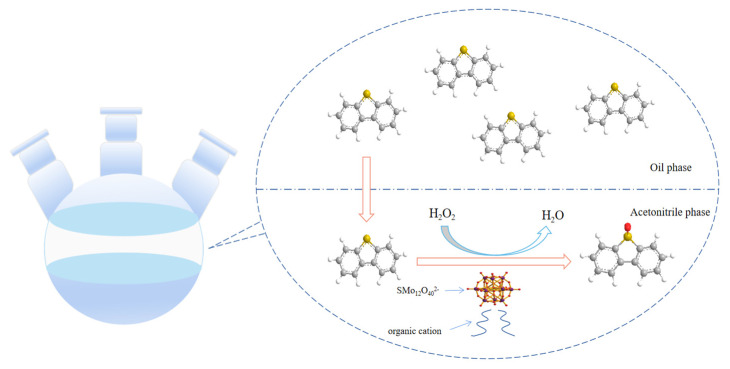
The ODS process of DBT using SMo_12_O_40_^2−^-organic hybrid catalysts.

Figure 13 shows the ODS process of DBT using SMo_12_O_40_^2−^-organic hybrid catalysts. In the ODS system, DBT in model oil was extracted into the acetonitrile phase. The cation of the amphiphilic hybrid catalyst is lipophilic, which can capture the DBT close to the oxidation active center, thereby increasing the contact area between the oxidant and sulfur compound and improving the desulfurization rate. When the SMo_12_O_40_^2−^ reacts with the oxidant H_2_O_2_, it will generate the peroxy-active species with a stronger oxidizing ability. This peroxy-active species is the real oxidant in the ODS reaction. DBT can be oxidized to DBT sulfone by the action of the oxidant. The oxidation product sulfone is dissolved in the acetonitrile phase to achieve separation from the oil.

## 3. Experimental

### 3.1. Synthesis of Catalysts

[TBA]_2_SMo_12_O_40_ was prepared by the method described in the literature [44]. A certain amount of Na_2_MoO_4_·2H_2_O (6.05 g, 25 mmol) was dissolved in 200 mL distilled water and stirred for 5 min at room temperature. NH_4_VO_3_ (0.6 g, 5.1 mmol) dissolved in H_2_SO_4_ (50 mL, 2 mol/L) was added and stirred for 5 min. Next, CH_3_COCH_3_ (250 mL) was added to the aforementioned system. After the mixture was stirred for 60 min at room temperature, tetrabutylammonium bromide (abbreviated as TBAB, 10 g, 31 mmol) was added. In order to complete metathesis, the above mixture was stirred continuously for 30 min. The precipitation was filtered and washed by distilled water and ethanol. The obtained solid catalyst was dried in a vacuum oven. The synthesis method of [BMIM]_2_SMo_12_O_40_ (6.8 g, 31 mmol) and [ODA]_2_SMo_12_O_40_ (12.2 g, 31 mmol) is similar to the above method, and only requires the replacement of the addition of TBAB with 1-butyl-3-methylimidazolium bromide (BMIMBr) or octadecyl trimethyl ammonium bromide (ODAB) in the reaction system.

### 3.2. Characterization

Infrared absorption spectra (IR) were performed for the 400–4000 cm^−1^ region on a Nicolet iS10 spectrometer (Thermo Fisher Scientific Co., Ltd., Waltham, MA, USA) with a resolution of 4 cm^−1^ and 32 scans. X-ray diffraction (XRD) patterns were recorded on a Brooke D8 advance power diffractometer (Brucker AXS Co., Ltd, Karlsruhe, Germany) in the range of 2θ from 5° to 60°. The SEM images were acquired by a 450 FEG electron microscope (United States FEI Co., Ltd., Hillsboro, OR, USA). Thermogravimetric analysis was carried out using a TG 209F3 thermal analyzer (Germany NETZSCH Co., Ltd., Selb, Germany) in the temperature range between 30 °C and 600 °C with a heating rate of 10 °C min^−1^ under nitrogen. X ray photoelectron spectrometric (XPS) were analyzed by a Thermo Fischer Escalab 250Xi spectrometer (Thermo Fisher Scientific Co., Ltd.).

### 3.3. Oxidative Desulfurization Process for Model Oil

The model oil is prepared by dissolving a certain amount of DBT (BT or 4,6-DMDBT) in 60 mL of n-octane. The initial sulfur content of the model oil is 500 ppm. The ODS system employs H_2_O_2_ as the oxidant and acetonitrile as the extractant. In a typical ODS experiment, 60 mL model oil with a sulfur content of 500 ppm, 60 mL acetonitrile, a certain dosage of catalyst, and H_2_O_2_ were added into a 250 mL three-neck flask. The three-neck flask was placed in a water bath with magnetic stirring. In order to reduce the evaporation of oil at different temperatures, the middle port of the three-neck flask was connected with a condenser tube and the left and right ports are plugged with plugs. Samples were taken from the upper oil phase at a certain time. The sulfur content of the sample was analyzed in a WK-2E microcoulometer. The conversion rate of DBT (BT or 4,6-DMDBT) was calculated using the following formula.

Conversion (%) = (C_0_ − C_t_)/C_0_ × 100%

where C_0_ is the initial sulfur content in oil, and C_t_ is the sulfur content at the different samples.

## 4. Conclusions

This paper presented an effective method for the deep desulfurization of model oil using the amphipathic SMo_12_O_40_^2−^-organic hybrid catalysts at room temperature. The prepared catalysts had a favorable desulfurization performance for sulfur compounds. Under optimized reaction conditions, when using [TBA]_2_SMo_12_O_40_ as a catalyst, the DBT conversion rate reached 99.89% in only 10 min, and when [BMIM]_2_SMo_12_O_40_ was employed as a catalyst, the DBT can achieve 99.81% removal within 15 min. The [ODA] _2_SMo_12_O_40_ catalyst can make the DBT conversion rate reach 99.47% within 60 min. The catalysts also have an excellent removal effect on BT and 4,6-DMDBT. The oxidation activity of the sulfur compound is affected by the electron density and steric hindrance. The desulfurization results of catalysts show that DBT is more easily oxidized than BT and 4,6-DMDBT. Under the condition of [ODA]_2_SMo_12_O_40_ as a catalyst, 4,6-DMDBT is easier to remove than BT, which is different from the desulfurization performance of the other two catalysts. The reason is that it is easier for the long alkyl chain to wrap up the larger molecule of 4,6-DMDBT close to the active center. The recycle performance of catalysts were investigated. The [TBA]_2_SMo_12_O_40_ immobilized in the acetonitrile phase was still reactive after the reaction, and can be reused at least five times for ODS. [BMIM] _2_SMo_12_O_40_ behaves as a heterogeneous catalyst in the ODS process, and can be recovered by simple filtration. [BMIM]_2_SMo_12_O_40_ still maintains excellent activity after five ODS cycles. When [TBA]_2_SMo_12_O_40_ and [BMIM]_2_SMo_12_O_40_ are applied to real diesel, they can achieve 99% and 98% desulfurization rates by ODS twice, respectively, indicating that the [SMo_12_O_40_]^2−^-organic hybrid catalysts have a favorable application prospect in industry. The ODS system using an SMo_12_O_40_^2−^-based catalyst provides a simple, mild and fast approach for deep desulfurization. However, the preparation cost of an SMo_12_O_40_^2−^-based catalyst is comparatively approximately 20% higher compared to that of the current commercial catalyst, and further R&D is needed for an SMo_12_O_40_^2−^-based catalyst with regard to laboratory and industrial applications.

## Figures and Tables

**Table 1:** Desulfurization performance of catalysts in a previous work and in this study.

Catalyst	Sulfur Compounds	Conversion (%)	Reaction Conditions	Reference
PMo_12_@UiO-67	DBT	99.50%	O/S = 3.1, 50 °C	[52]
PW12@UiO-67	DBT	99.50%	70 °C	[53]
[Bmim]_3_PMo_12_O_40_	DBT	100%	O/S = 3, 60 °C	[54]
[(C_8_H_17_)_3_NCH_3_]_3_PMo_12_O_40_/γ-Fe_2_O_3_@SiO_2_@mSiO_2_	DBT	100%	120 °C	[55]
[C_8_quin_]4_Mo_8_O_26_	DBT	99.2%	60 °C	[56]
[TBA]_2_SMo_12_O_40_	DBT	99.89%	O/S = 15, 60 °C	This work
[TBA]_2_SMo_12_O_40_	BT	97.06%	O/S = 15, 60 °C	This work
[TBA]_2_SMo_12_O_40_	4,6-DMDBT	95.20%	O/S = 15, 60 °C	This work

**Table 2:** Desulfurization performance of catalysts in actual diesel.

Catalyst	Initial Sulfur Content	By ODS	By ODS Twice	Total Desulfurization Rate
[TBA]_2_SMo_12_O_40_	514.53	93.17	20.18	96.08%
[BMIM]_2_SMo_12_O_40_	514.53	115.2	41.52	91.93%

## Data Availability

Not applicable.
